# The impact of body mass index (BMI) on satisfaction with work life: An international BODY‐Q study

**DOI:** 10.1111/cob.12527

**Published:** 2022-05-16

**Authors:** Amalie L. Jacobsen, Claire E. E. DeVries, Lotte Poulsen, Danny Mou, Anne F. Klassen, Andrea L. Pusic, Dennis J. S. Makarawung, Marinus J. Wiezer, Ruben N. van Veen, Jens A. Sørensen

**Affiliations:** ^1^ Research Unit for Plastic Surgery Odense University Hospital Odense Denmark; ^2^ OPEN, Open Patient data Explorative Network Odense University Hospital Odense Denmark; ^3^ Department of Surgery Brigham and Women's Hospital Boston Massachusetts USA; ^4^ Department of Surgery OLVG West Amsterdam The Netherlands; ^5^ Department of Pediatrics McMaster University Hamilton Ontario Canada; ^6^ Department of Surgery St. Antonius Hospital Nieuwegein The Netherlands; ^7^ Department of Plastic Surgery University of Southern Denmark Odense Denmark

**Keywords:** BMI, bariatric surgery, health‐related quality of life, patient‐reported outcome measures, work life

## Abstract

Obesity is a global health issue known to have a major influence on health‐related quality of life (HR‐QOL). HR‐QOL is a concept evaluating physical and psychological health. Work life can impact HR‐QOL in people with obesity. The aim of this study was to measure the association between body mass index (BMI) and satisfaction with work life. This study included participants from an international multicenter field‐test study of BODY‐Q scales. Recruitment took place at hospitals in Denmark, The Netherlands and USA between June 2019 and January 2020. The BODY‐Q Work Life scale was used to measure work life satisfaction. The difference between BMI groups and work life satisfaction was examined using one‐way analysis of variance. Multivariable linear regression analysis was used to examine the association between BMI and work life satisfaction, adjusted for significant confounders. Of 4123 participants, 2515 completed the BODY‐Q Work Life scale. BMI groups showed significant difference in work life satisfaction (*p* < .0001). The Work Life scale mean score was 77.6 for the normal BMI group, 78.5 for the overweight group and 75.0, 68.9 and 63.8 for Class 1, 2 and 3 obesity, respectively. Furthermore, BMI was significantly associated with satisfaction with work life (adjusted regression coefficient −.962, *p* < .0001). Higher BMI was associated with lower work life satisfaction. This finding suggests that a reduction in BMI may have a positive influence on work life satisfaction in people with obesity.

**What is already know about this subject**

- Obesity is known to have negative impact on people's health‐related quality of life.
- Obesity can result in weight‐related discrimination and has been shown to be associated with increased absenteeism and reduced work performance.
- Bariatric surgery (BS) is known to be the most effective treatment for obesity, resulting in weight loss and improved health‐related quality of life.

**What this study adds**

- Increasing BMI is shown to have a direct negative correlation to work‐related quality of life.
- Work‐related quality of life is shown to be better in people with obesity after they have had BS.
- This research suggests that BS could be beneficial for work‐related quality of life in people with obesity.

## 1 BACKGROUND

Obesity is an important and increasing global health issue. At least 2.8 million people die from obesity‐related medical conditions every year.^1^, ^2^ The correlation between obesity and chronic diseases has been well‐documented.^3^, ^4^ In addition, obesity is known to have major impact on people's psychological well‐being.^5^, ^6^, ^7^ A review examining how health‐related quality of life (HR‐QOL) is related to body mass index (BMI) has shown that higher BMI is associated with diminished HR‐QOL.^8^ An important aspect of HR‐QOL of people with obesity is work‐related problems, especially job security.^9^ Obesity can result in weight‐related discrimination and has been shown to be associated with increased absenteeism and reduced work performance, which may impose an economic liability to the workplace.^10^, ^11^ Furthermore, it has been suggested that obesity causes higher unemployment rates and lower job satisfaction.^12^, ^13^, ^14^

Bariatric surgery (BS) is known to be the most effective treatment for obesity, resulting in weight loss and improved HR‐QOL.^15^, ^16^, ^17^, ^18^ Thus, BS may be a key factor to alleviate some of the challenges that negatively influence people with obesity in their work life. Furthermore, it is known that BS is associated with a reduction in employment absence and an improvement in work productivity.^19^ Patients also reported significant improvements in mental and physical function related to work after BS and it is suggested that BS may improve the employment rate in people with severe obesity.^19^, ^20^, ^21^

Change and improvement related to specific treatments is best assessed by condition‐specific well developed and psychometrically sound patient‐reported outcome measures (PROMs).^22^ PROMs are questionnaires that allow patients to report their own health condition and HR‐QOL without a clinician's interpretation.^23^ Systematic reviews and meta‐analysis of randomised trials examining the relationship between obesity, weight loss, and HR‐QOL described that a limitation of research is the inconsistent use of generic and disease‐specific PROMs.^24^, ^25^ In a review that addressed the quality of PROMs for BS and body contouring surgery (BC), de Vries et al.^26^ found that the BODY‐Q demonstrated the strongest validation evidence for use in BS and BC patients.

The BODY‐Q is a condition‐specific, validated PROM that measures HR‐QOL, appearance and experience of health care for use in obesity, BS and BC. The BODY‐Q was developed according to guidelines for adequate development of PROMs.^27^ The development process included a literature review, 63 patient interviews, 22 cognitive patient interviews and input from 9 experts.^28^ An international sample of 734 participants completed the BODY‐Q, and Rasch measurement theory analysis was used to examine its psychometric properties.^29^, ^30^ Recently, five new BODY‐Q scales were developed and field‐tested in an international sample of 4004 participants.^31^ These scales were designed to measure eating‐related concerns for people seeking treatment for weight loss and included a scale measuring weight‐related work life issues. Each BODY‐Q scale is independently functioning, which enables the user to select the scales that suit their research or clinical purpose. The new scales were developed following the same rigorous guidelines as the original scales, and evidence of their validity and reliability is reported elsewhere.^31^

The aim of this study was to measure the association between BMI and satisfaction with work life using the BODY‐Q Work Life scale.

## 2 METHODS

This study used the BODY‐Q Work Life scale field‐test study dataset. The BODY‐Q Work Life scale will be referred to in the rest of the article as the Work Life scale. Data for this new BODY‐Q scale were collected as part of an international multicenter prospective cohort study that included Odense University Hospital and Hospital of Southwest Jutland in Denmark, Brigham and Women's Hospital in USA, Boston, MA. and OLVG West and St. Antonius Hospital in The Netherlands. The sample also included participants in Canada and USA recruited from the online research platform Prolific Academic.

Each site, except Denmark, received approval from the respective ethics committee for the collection of data. In Denmark, as this study was based on questionnaire data, approval from The Regional Committee on Health Research Ethics for Southern Denmark was not needed. The project was included on the list of health research within the Region of Southern Denmark.

Eligible participants were over 18 years old with the ability to provide informed consent and understand the primary language of the recruitment site.

### 2.1 Data collection

#### 2.1.1 Odense University Hospital and Hospital of Southwestern Jutland, Denmark

Recruitment took place between December 2019 and January 2020 and was based on the Danish BODY‐Q database including patients pre‐ and post‐BS who were currently, or had earlier been, in a treatment course at a Danish hospital. These patients have previously filled out the BODY‐Q and thereby contributed data for earlier studies on bariatric patients.^32^, ^33^ The patients included in the database who consented to be contacted again were sent the new BODY‐Q scales for testing. Up to three reminder emails were sent to nonrespondents.

#### 2.1.2 OLVG West and St. Antonius, The Netherlands

Recruitment took place between June 2019 and December 2019 and patients included were involved in an ongoing study at OLVG West and St. Antonius Hospital. Patients who met the inclusion criteria were sent an email with an individual URL to use to complete the BODY‐Q online in a secure web‐based programme (Castor EDC^34^). Nonresponders were sent up to two email‐reminders.

#### 2.1.3 Brigham and Women's Hospital, USA

Recruitment took place between July 2019 and January 2020. Patients who met the inclusion criteria were approached in the BS clinic and invited to participate in the study. Those who consented were asked to fill out the BODY‐Q on a tablet, where data entered a secure web‐based programme (REDCap^35^, ^36^). Patients who lacked time to finish the questionnaire in the clinic were sent the link via email to finish at home. Nonresponders were sent up to two reminder emails.

#### 2.1.4 Prolific research platform

Prolific participants were recruited from USA and Canada in September 2019. Prolific Academic^37^ is an online platform that collects data from people who agree to take part in research questionnaire surveys. Prolific users were invited to participate through an email invitation from the organisation. Participation in the study included the completion of original and new BODY‐Q scales. After providing online informed consent, participants were directed to the questionnaire via an URL linked to a REDCap survey hosted at Brigham and Women's Hospital. The Prolific participant group will be referred to in the rest of this article as non‐clinical participants. We sent an additional survey that asked Prolific participants if they had BS or other weight loss treatments in March 2020.

### 2.2 Outcome variables

Participants who had worked in a job with coworkers 3 months prior to completing the BODY‐Q survey were eligible to complete the Work Life scale. The Work Life scale includes 10 items with a series of statements that ask about the following issues in the workplace; feeling accepted, listened to, treated equally, standing up for yourself, having equal opportunities, having confidence, eating around coworkers, confidence at social events, feeling good and comfortable about weight. Each item has four response options that measure agreement (i.e., definitely disagree to definitely agree). The raw score for this scale was converted into a Rasch transformed score (0–100), with higher scores indicating higher work life satisfaction. In addition to the Work Life scale, data for the following demographic variables were collected: age, gender, education level, part‐time or full‐time employment status, time since any weight loss treatment, medical or surgical weight loss treatment, type of weight loss surgery, country of recruitment and clinical or non‐clinical participant group. Demographic characteristics are shown in Table 1.

**TABLE 1:** Sample characteristics and results from one‐way ANOVA and *t*‐test analysis

Sample (*n* = 2515)		Number of participants	Percent	Work Life scale mean score (95% confidence intervals)	Standard deviation	*p* Value
Country (*n* = 2515)	USA	1607	63.9%	70.8 (69.9–71.7)	18.6	<.0001
	Netherlands	256	10.2%	83.8 (81.7–85.8)	17.0	
	Denmark	620	24.6%	79.2 (77.6–80.8)	20.4	
	Canada	32	1.3%	67.3 (61.6–73.0)	15.8	
Participant group (*n* = 2515)	Clinical	1328	52.8%	77.6 (76.7–78.8)	19.7	<.0001
	Non‐clinical	1187	47.2%	70.1 (69.0–71.1)	18.3	
BMI (*n* = 2462)	Normal (18.5–24.9)	599	24.3%	77.6 (76.14–79.1)	18.9	<.0001
	Overweight (25–29.9)	756	30.7%	78.5 (77.1–79.9)	19.3	
	Class 1 obesity (30–34.9)	446	18.1%	75.0 (73.2–76.7)	18.8	
	Class 2 obesity (35–39.9)	306	12.4%	68.9 (66.9–70.9)	17.9	
	Class 3 obesity (≥40)	355	14.4%	63.8 (61.9–65.6)	17.6	
Age (years) (*n* = 2496)	18–29	442	17.7%	68.8 (67.0–70.6)	19.2	<.0001
	30–39	564	22.6%	72.6 (71.0–74.1)	18.8	
	40–49	652	26.1%	76.1 (74.6–77.6)	19.2	
	50–59	623	25.0%	76.8 (75.3–78.3)	19.2	
	>60	215	8.6%	76.2 (73.4–79.0)	20.6	
Gender (*n* = 2487)	Female	1670	67.1%	74.8 (73.9–75.6)	19.7	.031
	Male	817	32.9%	74.0 (71.8–74.3)	18.8	
Education (*n* = 2469)	Attending high school/not graduated high school	66	2.7%	77.0 (71.3–82.7)	23.1	<0.0001
	High school diploma	326	13.2%	73.4 (71.1–75.7)	21.3	
	Some college trade or university degree	644	26.1%	75.5 (74.0–77.0)	19.6	
	Completed college trade or university degree	977	39.6%	74.3 (73.1–75.5)	19.2	
	Some masters or doctoral degree	132	5.3%	70.6 (67.3–73.9)	19.0	
	Completed masters or doctoral degree	315	12.7%	71.9 (70.0–73.8)	17.2	
	Other	9	0.4%	75.6 (58.5–92.7)	22.3	
Employment status (*n* = 2510)	Full‐time	1708	68.0%	74.0 (73.1–74.9)	19.5	.749
	Part‐time	802	32.0%	74.5 (73.1–75.8)	19.4	
Time since surgery (*n* = 1228)	Pre operation	218	17.7%	64.7 (62.4–67.0)	17.2	<.0001
	First year after operation	336	27.4%	79.9 (77.9–81.9)	18.9	
	1–2 years after operation	312	25.4%	84.4 (82.5–86.3)	17.1	
	3 or more years after operation	362	29.5%	81.8 (79.9–83.6)	18.8	
Weight loss treatment (*n* = 1483)	Medical	248	16.7%	66.2 (63.9–68.4)	18.0	<.0001
	Surgical	1235	83.3%	78.9 (77.9–80.0)	19.3	
Weight loss surgery type (*n* = 1020)	Gastric banding	36	3.5%	76.4 (70.3–82.4)	17.9	<.0001
	Gastric bypass	610	59.8%	84.0 (82.6–85.4)	17.7	
	Sleeve gastrectomy	369	36.2%	78.8 (76.8–80.6)	19.1	
	Other	5	0.5%	76.4 (56.9–95.9)	15.7	

Abbreviations: ANOVA, analysis of variance; BMI, body mass index.

### 2.3 Statistical analysis

Data analysis was performed using IBM SPSS Statistics for Windows.^38^ BMI was calculated from reported weight and height.

Patient characteristics were described as percentages or the mean ± standard deviation (SD). Distribution of the Work Life scale (normality) was evaluated by histograms. To examine the difference in Work Life scale outcome in relation to the demographic variables of the study population, we performed independent *t*‐test for dichotomous variables or one‐way analysis fo variance for categorical variables. A multivariable linear regression model was used to assess the association between BMI and Work Life scale outcome which was the main objective of this study. Before analysis, normality of BMI, age and time since treatment were evaluated with histograms, and continuous variables that were not normally distributed were rescored as categorical variables. We evaluated whether all assumptions for regression analysis were met for further analysis. In addition to the crude analysis, the model was adjusted for baseline variables that were significant confounders. The continuous potential confounders included BMI and age. The categorical variables that were used as dummies included education (high school, college, masters), country (USA/Canada, the Netherlands, Denmark) and time since treatment (pre‐operative, first year, 1– 2 years, 3 years or more). The dichotomous potential confounders included gender (male vs. female), medical or surgical weight loss treatment, type of weight loss surgery (sleeve gastrectomy vs. gastric bypass) and clinical or non‐clinical participant group. Findings were considered statistically significant for a two‐tailed significance level of *p* < .05. Confounders were identified by a 10% or greater difference between the values of the regression *β* in the crude and adjusted analysis of association.

To detect effect modification of the clinical or non‐clinical group, further analyses were performed with the interaction between clinical or non‐clinical group and BMI (a *p* < .05 indicated an interaction effect). If there was a significant interaction effect, a stratified analysis was performed following the same method described above.

## 3 RESULTS

**FIGURE 1:**
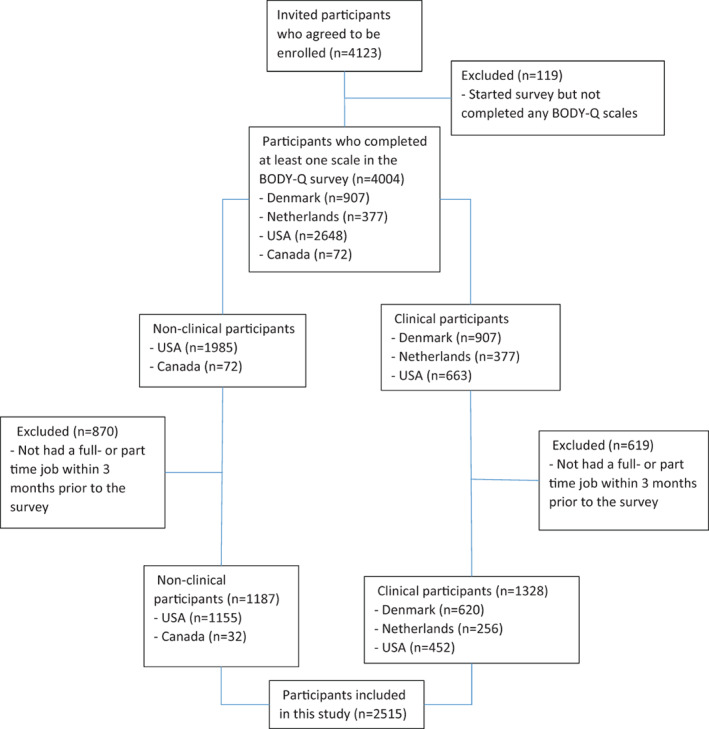
Flow diagram illustrating study enrollment process

Of the participants invited to complete the survey 64% agreed to be enrolled in the study. Overall, a total of 4123 people participated in the study. Of the enrolled participants, 2515 (61%) were working full or part‐time in the past 3 months and filled out the Work Life scale. The enrollment rate for the invited participants varied by recruitment country as follows: Denmark 59% (*n* = 620), The Netherlands 62% (*n* = 256) and USA 73% (*n* = 1607). Figure 1 illustrates the study enrollment process.

### 3.1 Demographics

The majority of participants (*n* = 756, 30.3%) were overweight (i.e., BMI 25–30). Most participants were female (*n* = 1670, 67.1%) and clinical rather than non‐clinical participants (*n* = 1328, 52.8% vs. 1187, 47.2%). The distribution of participants within the different age groups were similar for age groups 30–39, 40–49 and 50–59 (*n* = 564, *n* = 652, *n* = 623) but differed for the 18–29 and ≥60 age groups (*n* = 442, *n* = 215). Of the participants who received treatment, more received surgical rather than medical treatment (*n* = 1235, 83.3% vs. *n* = 248, 16.7%). The mean BMI was 30.9 kg/m^2^ SD 8.3, mean age was 42.9 years SD 12.2. The median time since treatment was 10 months, interquartile range 6. Other participant characteristics are shown in Table 1.

### 3.2 BODY‐Q Work Life scale

The overall mean Work life scale score for all participants was 74.1 (0–100). Table 1 shows the Work Life scale mean scores for the different demographic characteristics of the sample. Significant differences were found for the following variables: country, participant group, BMI classification group, age group, gender, type of weight loss treatment, type of weight loss surgery and time since surgery group.

**FIGURE 2:**
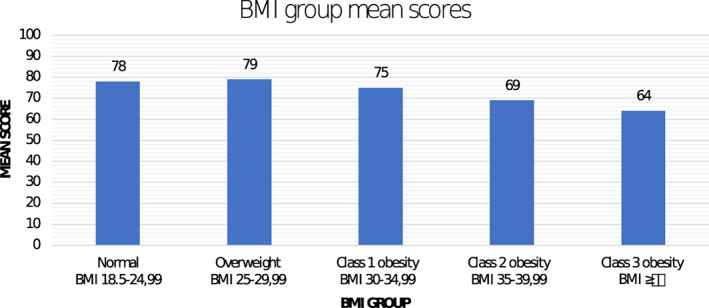
BODY‐Q Work Life scale mean scores divided by BMI groups. BMI, body mass index

A significant difference in Work Life scale mean score was found between countries. Participants from the Netherlands reported the highest mean score followed by Denmark, the USA and Canada. The patients recruited in the clinic had a higher Work Life scale mean score compared to the non‐clinical participant group. As Figure 2 illustrates, the mean score for the Work Life scale was lower in higher BMI groups. Age groups from 40 years and up had similar Work Life scale mean scores, but the younger age groups scored lower. Women showed a slight tendency towards higher Work Life scale mean score than men (*p* = .031). Time since surgery showed a difference in Work Life scale mean scores with the lowest score found in the pre‐operative group, whilst the highest score was found in the group recruited 1–2 years after surgery (*p* < 0.0001). The mean score on the Work Life scale was higher in the BS group than the medical weight loss group (*p* < 0.0001). By type of surgery within the BS group, patients who had gastric bypass reported the highest mean score.

There was no difference found in the Work Life scale mean score for employment status. Education showed a significant difference between groups (*p* < .0001) but seemed to differ arbitrarily and did not show a clear tendency associated with higher or lower education level.

### 3.3 Multivariable linear regression analysis

In the multivariable linear regression model presenting the association between BMI and work life satisfaction, the crude regression coefficient was −.539, 95% confidence interval (CI) −0.677 to 0.497, *p* < .0001. After adjusting the multivariable linear regression model for significant confounders (age, country [dummy variables USA/Canada, Netherlands, Denmark] and pre‐operative vs. post‐operative), the regression coefficient was −0.736, 95% CI −0.892 to −0.580, *p* < .0001. Effect modification of clinical versus non‐clinical participant group was detected, so we performed further stratified analysis. For the clinical group only we found the crude regression coefficient was −0.983, 95% CI −1.098 to −0.869, *p* < .0001 and adjusted for significant confounders (pre‐operative vs. post‐operative) the regression coefficient was −0.872, 95% CI −1.004 to −0.740, *p* < .0001. For the non‐clinical group only we found the crude regression coefficient was −0.611, 95% CI −0.762 to −0.459, *p* < .0001 and adjusted for significant confounders (age) the regression coefficient was −0.699, 95% CI −0.0853 to −0.546, *p* < 0.0001.

Table 2 shows adjusted and crude models.

**TABLE 2:** Results of the linear multivariate linear regression analysis

Collected results					
	Model	Regression coefficient BMI^a^	95% Confidence interval		*p* Value
BODY‐Q Work Life impact	Crude	−0.587	−0.677	−0.497	<.0001
	Adjusted^a^	−0.736	−0.892	−0.58	<.0001

Clinical group only					
	Model	Regression coefficient BMI^b^	95% Confidence interval		*p* Value
BODY‐Q Work Life impact	Crude	−0.983	−1.098	−0.869	<.0001
	Adjusted^b^	−0.872	−1.004	−0.74	<.0001

Non‐clinical group only					
	Model	Regression coefficient BMI^c^	95% Confidence interval		*p* Value
BODY‐Q Work Life impact	Crude	−0.611	−0.762	−0.459	<.0001
	Adjusted^c^	−0.699	−0.0853	−0.546	<.0001

^a^ Adjusted for age, country (dummy variables USA/Canada, Netherlands, Denmark) and pre‐operative vs. post‐operative.

^b^ Adjusted for pre‐operative vs post‐operative.

^c^ Adjusted for age.

## 4 DISCUSSION

BS is the most effective weight loss treatment and improves overall HR‐QOL, work capacity and employment rates.^21^, ^39^, ^40^, ^41^, ^42^, ^43^ However, because of the lack of rigorously developed instruments for measuring outcomes of BS on issues reported from the patients themselves, little is known about the specific correlation between BMI and work life satisfaction, or the effect of BS on this relationship.^26^ The results from this study, in conjunction with current literature, imply that further workplace benefits can be obtained from weight loss.

The BODY‐Q Work Life scale represents a new generation PROM developed specifically for use in patients with obesity and patients undergoing weight loss treatments. Overall, our findings confirmed that BMI was associated with work life satisfaction. When divided into BMI categories there was a slight increase in Work Life scale mean score from the normal BMI group to the overweight BMI group and then showed a prominent decrease in the Class 1, 2 and 3 obesity BMI groups. However, the multivariable linear regression model showed a clear negative association between increase in BMI and Work Life scale mean score. After adjusting for significant confounding factors, we found that for every one‐point BMI increase, the score on the Work Life scale decreased with 0.736. The ascent in BMI group from, e.g., BMI 26 which is overweight to BMI 38 which is Class 2 obesity would thus be associated with a decrease in work life satisfaction of almost 10 points, on a scale from 1 to 100. BMI over 40 is a clear indication for BS^44^, ^45^ and according to our analysis it is suggested that this patient group can have a work life satisfaction almost 20 points (on a scale from 1 to 100), lower than a person with a BMI in the normal BMI group (18.5–25).

Effect modification of clinical versus non‐clinical group was detected, so we performed further stratified analysis and conducted a multivariate regression model for the two groups individually. This analysis showed that both groups had a significant negative association between increase in BMI and Work Life scale mean score, but the association was more pronounced in the clinical group compared to the non‐clinical group. This could indicate that people undergoing bariatric treatment are more likely have their work life satisfaction negatively affected by a BMI increase, which could have been a contributing factor for them to seek bariatric treatment. The difference in the two groups could also possibly indicate that bariatric treatment has had a positive influence on the work life satisfaction on the clinical group.

The fact that the Work Life scale mean score was found lowest in the pre‐operative group and then increased substantially in the post‐operative groups suggests that BS induced a weight loss with major effect on work life satisfaction. The Work Life scale mean score peaks in the group who were 1–2 years after BS. This correlates with literature, where a study shows lowest BMI between 18–24 months after BS in patients who have undergone gastric bypass.^46^ In the same study, a weight regain is shown 24 months after BS which correlates with the slight decrease in Work Life scale mean score found in our study in the 3 years or more since surgery group. Gastric bypass surgery has shown greater and more sustainable weight loss than gastric banding and sleeve gastrectomy.^47^, ^48^ This could possibly explain the higher Work Life scale mean score in the gastric bypass group in our study, and this could also support the suggestion that lower BMI increases Work life satisfaction.

To our knowledge this is the first study using evidence‐based PRO data to elucidate the relationship between BMI and work life satisfaction. This knowledge can be useful for clinicians in counselling patients who seek bariatric treatment. The decision to choose one bariatric treatment over another is complex and can neither be based on the patient's wishes nor the clinician's advice alone. Optimal shared decision is accomplished in the pre‐operative counselling process by correctly informing the patients of what to expect from BS. This is the basis for a judicious united decision. Our analysis showed a clear association between lower BMI and higher work life satisfaction. This finding suggests that weight loss might improve individuals' work life satisfaction. A longitudinal study is needed to illuminate the causality of weight loss and improvement in the work life satisfaction in order to measure the amount of change in work life satisfaction before and after BS.

There are several limitations to this study. Overall, the cross‐sectional design of the study poses a limitation as the causality between BMI and work life satisfaction can only be conjecture. Within the study we find following limitations. First, the sample size from the contributing countries varied significantly. The number of respondents might have been biased by having different recruiting protocols at the sites of the contributing countries. The lowest Work Life scale mean score was found in the Canadian participants which also presented the smallest sample size and may therefore not be representative. Second, the non‐clinical participant group was anonymous, and we know little about them and their motives for participating. A significant part, 32%, of the total study‐population were not full‐time employed when taking the survey and the distribution of employment between the clinical and non‐clinical participant group is unknown. Third, the type of employment was unknown in this study and may play an important role in work life satisfaction for people with weight‐related issues. Even though we controlled for the limitations regarding age, country and pre‐ or post‐operative group in our regression analysis—other unknown variables may, however, still bias the result. Finally, we assessed the BMI of the participants from self‐reported weight and height and this may lead to information bias. The use of BMI to determine the patient's level of obesity, can in itself be considered a limitation as it is not the most precise assessment for adiposity. Furthermore selv‐reported BMI can be influenced by many physical, psychological and social factors. Therefore, it is relevant to consider the potential effect bariatric treatment alone could have on body image and self‐perception and that this could possibly have affected the reported BMI. However, BMI is commonly used as it is the most accessible choice when collecting data from a questionnaire survey.^49^

## 5 CONCLUSION

Overall, our findings confirm that BMI is related to work life satisfaction. Our analysis showed that a lower work life satisfaction was associated with increased BMI. This finding supports that a decrease in BMI, alongside other HR‐QOL improving qualities, might have the potential to positively influence the work life of people with obesity. This result can be useful in the clinical guidance of people seeking BS and benefit shared decision‐making.

## CONFLICT OF INTEREST

Anne Klassen and Andrea Pusic are co‐developers of the BODY‐Q and, as such, receive a share of any licence revenues as royalties based on their institutions inventor sharing policy. Other authors declare no conflicts of interest.
